# Image-based temporal profiling of autophagy-related phenotypes

**DOI:** 10.1080/27694127.2025.2484835

**Published:** 2025-04-08

**Authors:** Nitin Sai Beesabathuni, Neil Alvin B. Adia, Eshan Thilakaratne, Ritika Gangaraju, Priya S. Shah

**Affiliations:** a Department of Chemical Engineering, University of California, Davis, California; b Department of Microbiology and Molecular Genetics, University of California, Davis, California

**Keywords:** Autophagy, autophagy flux, cargo degradation, fluorescence microscopy, live cell imaging, morphological features, rapamycin, temporal profiling, wortmannin

## Abstract

Autophagy is a dynamic process critical in maintaining cellular homoeostasis. Dysregulation of autophagy is linked to many diseases and is emerging as a promising therapeutic target. High-throughput methods to characterise autophagy are essential for accelerating drug discovery and characterising mechanisms of action. In this study, we developed a scalable image-based temporal profiling approach to characterise ~900 morphological features at a single cell level with high temporal resolution. We differentiated drug treatments based on morphological profiles using a random forest classifier with ~90% accuracy and identified the key features that govern classification. Additionally, temporal morphological profiles accurately predicted biologically relevant changes in autophagy after perturbation, such as total cargo degraded. Therefore, this study acts as proof-of-principle for using image-based temporal profiling to differentiate autophagy perturbations in a high-throughput manner and has the potential identify biologically relevant autophagy phenotypes. Ultimately, approaches like image-based temporal profiling can accelerate drug discovery.

## Introduction

Macroautophagy (hereafter referred to as autophagy) is an intracellular process that recycles damaged and unwanted cellular components to maintain homoeostasis. In autophagy, specialised double-membraned structures called phagophores grow to surround cellular contents such as proteins and organelles to form autophagosomes. Following the fusion of autophagosomes with lysosomes to form autolysosomes, the cellular cargo is degraded by lysosomal enzymes. This recycling process generates free amino acids, fatty acids, and energy for biosynthetic pathways, and can turn over damaged proteins and organelles [1].

Autophagy is linked to many diseases and disorders [2–4]. For example, failure to clear damaged proteins and organelles from long-lived neurons results in cytotoxicity and neurodegeneration [5]. Upregulating autophagy can help cancer cells survive in nutrient-depleted environments [6]. Autophagy can also be co-opted during virus infection [7]. Given its role in disease, fine-tuning autophagy to reverse or prevent disease is of great interest [8,9].

Quantitative and holistic characterisation of autophagic activity is essential to modulating it therapeutically in disease. Microtubule-associated protein 1A/1B-light chain 3 (LC3) is a commonly used marker to monitor autophagy [10]. LC3 is often tagged with fluorescent molecules to quantify autophagy phenotypes [11]. Nevertheless, quantitative measurements of autophagy at a single cell level are primarily limited to autophagy vesicle count using fluorescence microscopy and image cytometry, or mean intensity of the cell using flow cytometry [11]. Although these measurements have enabled high-throughput, quantitative, and sensitive detection of autophagy, they fail to capture the autophagy state comprehensively. The two main reasons are 1) vesicle count and mean intensity alone do not inform the rate of flow of cargo through the pathway, often referred to as autophagy flux [11], and 2) other properties such as shape, size, distribution of autophagy vesicles over time, and whole cell morphological changes are often not analysed, leaving vast amounts of phenotypes uncharacterised.

We recently developed a quantitative framework to address the first challenge related to measuring the rate of cargo flow through the autophagy pathway [12,13]. Our approach expands on the quantitative steady-state autophagy flux analysis originally developed by Loos and colleagues [14]. We apply an instantaneous rate approach to measure the rate of each step in the pathway under dynamic conditions. By uncoupling measurements of the rate of autophagosome formation from the rate of autophagosome-lysosome fusion and autolysosome turnover, we can better distinguish between the direct mechanisms of action and long-term feedback loops in response to environmental perturbations (*e.g*., chemicals, nutrients, or infection). We can also quantify the absolute (as opposed to relative) total degradative capacity of this dynamic and adaptive system, which can assist in precisely fine-tuning autophagy flux. However, this approach involves destructive sampling, limiting the scalability of this method to characterise thousands of conditions in a high-throughput manner, even with automation.

Image-based profiling facilitates the simultaneous quantification of various morphological features at a single cell level. These morphological features provide information on the size, shape, and texture of visualised cells and sub-cellular components. Some features have a more direct physical interpretation, such as the area or intensity of a sub-cellular component in an image, while others are less straightforward, such as the correlation between pixel intensities within a cell or their distribution. Image-based profiling has been used to characterise small-molecule regulators and genetic modulators of the autophagy pathway [15–17]. These studies highlight the potential of using image-based profiling for high-throughput autophagy characterisation. However, they were performed at a single time point after fixing cells, limiting our understanding of dynamic changes in autophagy phenotypes.

To capture autophagy dynamics, we created an image-based *temporal* profiling platform to systematically characterise dynamics in autophagy-related morphological phenotypes in live cells. We investigated changes in morphological features following the addition of two well characterised small molecule autophagy modulators, rapamycin and wortmannin. We examined the key morphological features differentially modulated under various rapamycin and wortmannin treatments as a function of time. Using a random forest classifier, we identified the features with high importance that can differentiate these treatments. We also used a profile similarity approach to test the possibility of characterising autophagy regulation based on temporal profiles of morphological features. Compared to canonical measurements of autophagy vesicle dynamics (i.e. number of autophagic vesicles or LC3 abundance over time), the inclusion of additional morphological features in temporal profiles captured the total degradative capacity of cells more accurately. We used these temporal profiles to predict the impact of other autophagy modulators on degradative capacity. Integrating morphological features improves predictions compared to using autophagic vesicle number alone. Integrating dynamics through temporal analysis also provides more reliable predictions compared to predictions based on a single timepoint. Furthermore, the systematic nature of this approach provides predictions in an unbiased manner based only on the automated image acquisition and analysis pipeline. Overall, this study serves as proof-of-concept for using image-based temporal profiling to characterise autophagy modulation without onerous destructive sampling. This approach has the potential to map novel drugs to mechanisms of action and facilitate high-throughput drug characterisation by linking complex morphological responses to biologically relevant cellular outputs.

## Results

### Experimental setup and image-based profiling pipeline

**Figure 1.**
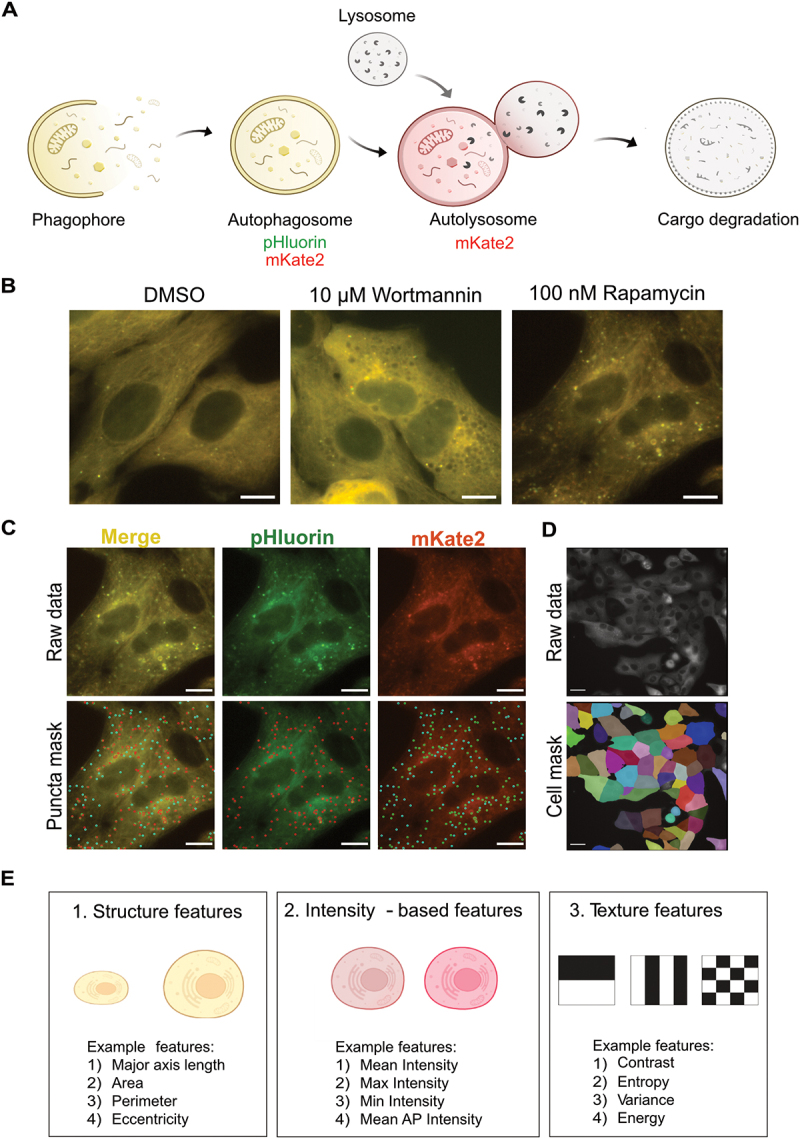
Experimental and image analysis pipeline to quantify autophagy-related phenotypes. (A) pHluorin-mKate2-LC3 was used for tracking autophagosomes and autolysosomes. (B) Representative images of change in morphology of cells after treatment with 100 nM rapamycin and 10 µM Wortmannin for 6 h. The scale bar represents 10 µm. (C) Representative images of spot detection tool for detecting autophagosomes (red spots in the pHluorin channel and green spots in the mKate2 channel) and autolysosomes (cyan spots in the mKate2 channel). Spots detected only in the pHluorin channel may represent fast-moving autophagosomes. Scale bar represents 10 µm. (D) Representative image of segmented cell mask used for tracking individual cells. Scale bar represents 20 µm. (E) Three main categories of morphological features were extracted at a cellular level as well as at an autophagy vesicle level.

We quantified various morphological features by reanalysing images that were previously collected to measure autophagy rates [12] via the pHluorin-mKate2-LC3 system [18] in Huh7 human hepatocarcinoma cells (Figure 1A). This approach is easily adaptable to other tandem fluorescent reporters fused to LC3 [19]. An illustrative image of the change in cellular morphology is shown in Figure 1B, where wortmannin treatment caused void-like regions in the cells, while rapamycin caused a decrease in the signal intensity of cells. Images of additional treatments and timepoints can be found in Figure S1 and Figure S2. We developed an image analysis pipeline to segment and track individual cells over time without a nuclear marker (Figure 1C-D). Along with autophagosome and autolysosome numbers, hundreds of features for three main morphological properties (structure, intensity, and texture) were quantified at a single cell level for whole cell (Cell), autophagosomes (AP), and autolysosomes (AL) (Figure 1E). Approximately 900 features for each cell were quantified for the entire time course of the experiment. A detailed pipeline is shown in Figure S3 and described in the methods.

### Image-based temporal profiling of morphological features during autophagy induction and inhibition

**Figure 2.**
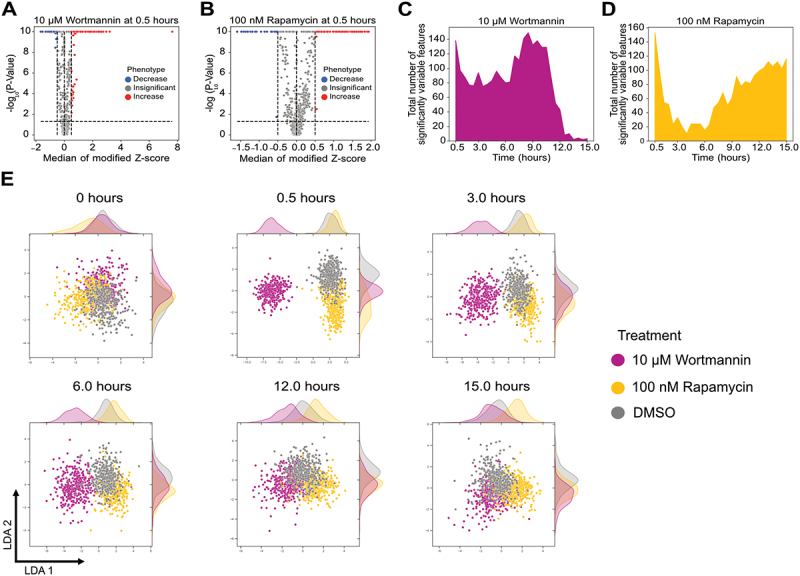
Temporal change in morphological features after rapamycin and wortmannin treatment. (A-B) Volcano plots of cellular features after 30 min of treatment with (A) 10 µM wortmannin and (B) 100 nM rapamycin, respectively. (C-D) Cellular features that varied significantly as a function of time for 10 µM wortmannin and 100 nM rapamycin, respectively (E) LDA of cells treated with DMSO, 100 nM rapamycin, and 10 µM Wortmannin at different time points. A minimum of 300 cells were analysed for each condition. Features that varied significantly with a median modified Z-score ≥ 0.5 at specific time points were used for generating the LDAs. For 0 h, features from 0.5 h were used.

We first characterised morphological changes in cells treated with high concentrations of rapamycin and wortmannin. We previously observed that rapamycin and wortmannin treatment increased and decreased overall autophagy flux, respectively, consistent with the known mechanisms of action for these small molecules [12]. We identified the features that varied significantly compared to DMSO-treated cells at each time point for both treatments. A table of the top 10 most variable figures is shown in Table S1. All features with a median modified Z score ≥ 0.5 with an adjusted P-value < 0.05 were considered significant. A representative analysis is shown at 0.5 h after treatment with 10 µM wortmannin and 100 nM rapamycin (Figure 2A-B). Identical analysis was performed on the same cells before treatment to confirm that a threshold of 0.5 removes any false-positive features that change significantly without treatment (Figure S4A-B). We compared measurements from our image analysis pipeline to those obtained from flow cytometry. Data from both methods showed a significant decrease in mean fluorescence intensity at 6 h following treatment with rapamycin (Figure S4C-D), confirming the consistency of the imaging pipeline with other methods commonly used for high-throughput screens of autophagy responses at a single cell level.

We next analysed the number of features that varied significantly with time (Figure 2C-D). Wortmannin and rapamycin treatment both led to dramatic increases in variable features immediately following treatment. Wortmannin-treated cells maintained this increase until ~12 h. Conversely, rapamycin-treated cells showed a rapid decrease following the initial peak and then a gradual increase in variable features that was maintained even at 15 h. We also analysed a lower concentration of wortmannin and wortmannin + rapamycin combination treatments using a similar approach and observed a concentration-dependent effect of wortmannin on cellular morphology dynamics (Figure S5A-C).

We next visualised the single-cell landscape for DMSO, wortmannin, and rapamycin treatment at multiple time points using uniform manifold approximation linear discriminant analysis (LDA) and projection (UMAP) and [20,21] (Figure 2E and S5D). LDA provided visually better separation compared to UMAP. While cells from different conditions were distributed generally evenly throughout a single LDA cluster before treatment, they formed distinct clusters based on treatment in as little as 30 min. Wortmannin-treated cells were more easily separated from DMSO-treated cells at all time points before 12 h compared to rapamycin. After 12 h, the wortmannin-treated cells clustered together with DMSO-treated cells which is consistent with the decrease in the differential features at later time points (Figure 2C). In conclusion, dynamic changes in morphological features can be used to visualise distinct cellular responses in an unsupervised manner.

### Feature importance in distinguishing rapamycin and wortmannin treatment using a random forest classifier

**Figure 3.**
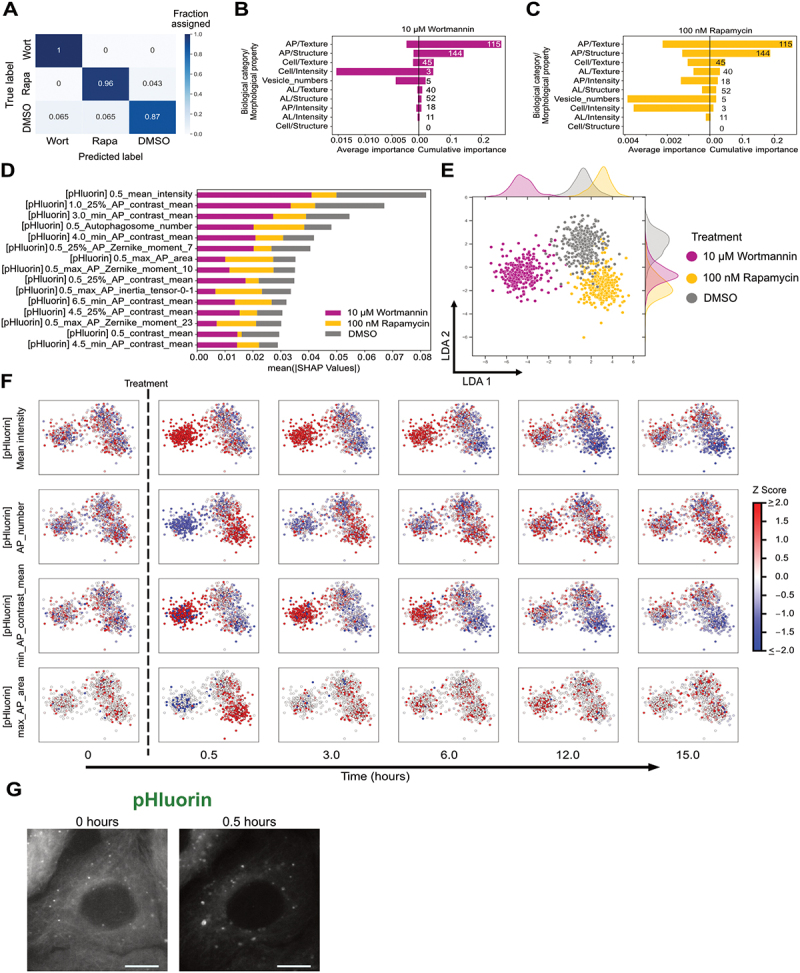
Identification of governing features to differentiate rapamycin and wortmannin treatments. (A) A confusion matrix to visualise the performance of the random forest model in classifying 10 µM wortmannin (Wort) and 100 nM rapamycin (Rapa) and DMSO treatments. A minimum of 70 cells for each condition were used for testing the accuracy of the model. (B-C) Cumulative and average importance of feature categories in classifying wort and rapa treatments, respectively. (D) The top 15 features with the highest mean absolute SHAP values. (E) LDA representation of individual cells constructed using variable features from all time points. A minimum of 300 cells were analysed for each condition. (F) Change in feature values at a single cell level with time after treatment with rapa and wort. These individual features were visualised over the LDA space. (G) Representative image of increase in autophagosome area after treating with rapa for 30 min. Green channel (pHluorin) images are shown in greyscale. Scale bar represents 10 µm.

Identifying governing features that distinguish one drug from another in the autophagy response would be a powerful tool for dissecting mechanism of action and discovering novel phenotypes. Towards this end, we constructed a random forest model to identify the primary feature set that could be used to differentiate rapamycin and wortmannin treatments (Figure 3A). The model was trained using all variable features after removing correlated features (see Materials and Methods). The model achieved an F_1_ score of 0.89 ± 0.033, which is a metric used to evaluate prediction model’s performance based on precision and recall (see Materials and Methods).

This random forest classifier was then examined using Shapley Additive exPlanations (SHAP) values to establish the importance of each feature in differentiating the treatments [22]. A higher absolute SHAP value represents a higher importance in classifying the treatments. We grouped the features based on biological category (Cell, AP, and AL) and morphological property (structure, intensity, and texture), then calculated the cumulative and average importance for each group in classifying wortmannin and rapamycin treatments (Figure 3B-C). Cumulative importance represents the sum of absolute SHAP values of all features in a group but may be biased towards groups with more features. Average importance represents the mean of absolute SHAP values of all features in a group, but may not capture subtle changes in many features for large groups. Vesicle numbers and Cell/Intensity groups had few features but contributed substantially towards the classification of both treatments indicated by the high average importance value. Autophagosome texture and structure groups had a high cumulative importance for both treatments but contained a large fraction of the variable features which contribute to the overall cumulative value. We analysed individual features that influenced the classification, with the top 15 shown in Figure 3D. The mean intensity of the cell (mean_intensity, taken from the GFP channel) at 0.5 h and autophagosome texture (min_AP_contrast_mean) were key features in differentiating wortmannin from DMSO treatment. Initial autophagosome number (AP_number) and autophagosome structural features such as maximum autophagosome area (max_AP_area) and maximum autophagosome Zernike moment (max_AP_Zernike_moment_10) were important in classifying rapamycin treatment.

To examine the impact of rapamycin and wortmannin on high importance features over time, we generated an LDA comprising all features and all time points (Figure 3E). The plot demonstrated a clear separation between cells subjected to the two treatments, possibly reflecting their different mechanisms of action and resulting impacts on autophagy rates. Interestingly, compared to snapshots of single timepoints (Figure 2E), the aggregated temporal data provided better overall separation of cells based on treatment and underlines the richness of temporal trajectories.

We visualised specific features on the LDA to understand changes based on treatment and time (Figure 3F). We chose mean_intensity, AP_number, minimum value of autophagosome contrast mean per cell (min_AP_contrast_mean), and max_AP_area because SHAP analysis indicated their importance in classifying autophagy response (Figure 3D). Wortmannin and rapamycin had opposing effects on initial mean_intensity and AP_number, consistent with their impacts on autophagy rates [12,23]. Autophagosome number and LC3 reporter mean intensity are also commonly used to quantify autophagy flux due to their intuitive nature [23], and underlines the consistency of our unbiased analysis with existing methods. In contrast, less intuitive morphological features were also informative in distinguishing treatments. Min_AP_contrast_mean, a strong indicator of wortmannin treatment, increased for a major fraction of wortmannin-treated cells. Max_AP_area increased after rapamycin treatment, indicating that larger autophagosomes form after rapamycin treatment (Figure 3G). This change may be explained by rapamycin inducing a rapid formation of autophagosomes. Thus, both intuitive and non-intuitive features contribute to treatment classification, suggesting that image-based temporal profiling could reveal new morphological biomarkers of autophagy perturbation.

To determine if this pipeline could be applied to autophagy measurements in different cell lines, we performed similar analyses on data from U2OS cells, an osteosarcoma cell line. We previously quantified autophagy flux and individual autophagy rates, and we observed similar trends between U2OS and Huh7 cells, though the magnitude of responses differed [12]. When applying image-based profiling to U2OS cells, our results showed similar trends but with reduced magnitude (Figure S6A-D). UMAP visualisation showed no separation of treatments at individual timepoints between treatments (Figure S6E), aggregating all timepoints gave reasonable classification by random forest model and slightly better cluster separation by UMAP (Figure S7). This is similar to our results with A549 cells (Figure 2E and 3E), reiterating the importance of temporal profiling. The different governing morphological features arising due to SHAP analysis (Figure S7) suggest that these differences arise from cell line-specific morphological behaviour. Thus, image-based profiling can classify different modes of autophagy perturbation in multiple cell lines, but may require cell line-specific analysis.

### Image-based temporal profiling of morphological features at different levels of autophagy induction

**Figure 4.**
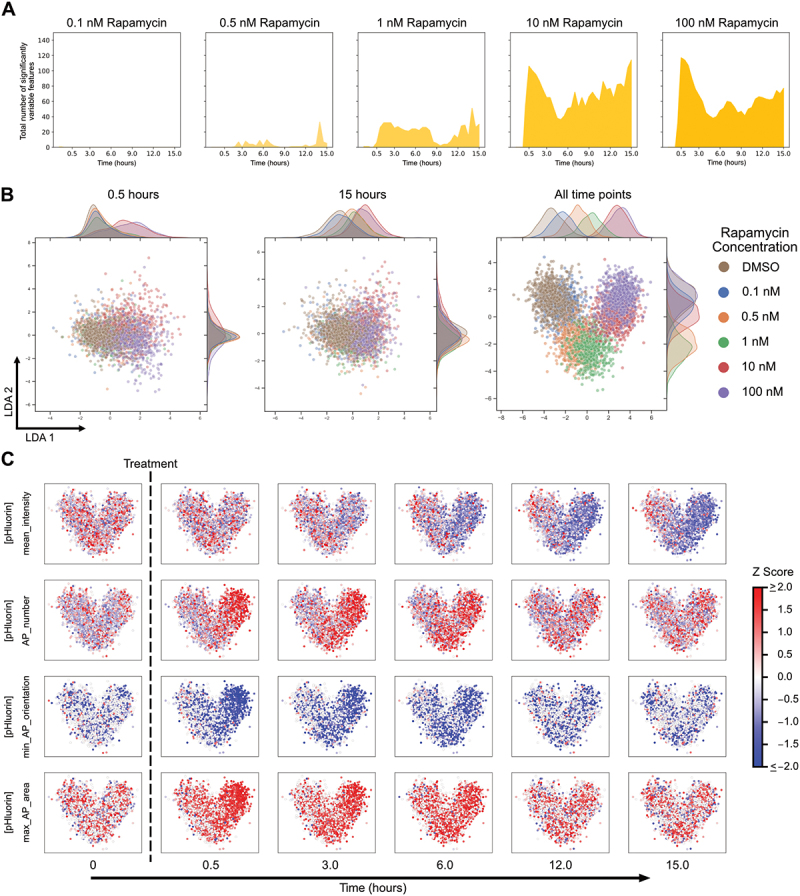
Morphological changes at different concentrations of rapamycin treatment. (A) Single cell features that varied significantly over time for the different rapamycin concentrations. (B) LDA representation of individual cells plotted using variable morphological features at all time points of treatment. (C) Changes in individual feature values at different time points after treatment with different rapamycin concentrations, visualised on the LDA space.

We next assessed the sensitivity of the system by characterising changes in cells treated with a range of rapamycin concentrations. We previously observed a concentration-dependent increase in overall autophagy flux [12]. We re-analysed this data to determine the number of features that varied significantly with time and observed a concentration-dependent increase in variable features (Figure 4A). We did not find great utility in applying a classifier to distinguish between treatment concentrations (data not shown). This was surprising given the dramatic concentration-dependent increase in the number of features varying significantly, and suggested that the dominant features are similar regardless of rapamycin concentration. We therefore analysed morphological features independent of SHAP analysis. We identified features that were highly correlated (or anticorrelated) with autophagosome number (Figure S8A). The identified features were also highly correlated (or anticorrelated) with one another (Figure S8A). The strong correlation between features could be one reason why a random forest classifier did not perform well. Given the strong correlations, the feature with the highest dynamic range, max_AP_Zernike_moment_16, was selected for analysis. Zernike moments quantify shape mathematically using orthogonal (non-redundant) pieces of information. A high Zernike moment means that the overall shape is more dominantly described by that particular moment [24]. max_AP_Zernike_moment_16 refers to the maximum value of the 16^th^ Zernike moment of autophagosomes and showed concentration-dependent behaviour like autophagosome number (Figure S8B). While this feature was slightly more sensitive than autophagosome number at earlier time points, at later time points it was less sensitive (Figure S8C-D).

We next visualised the single cell morphological landscape to study the concentration-dependent effects of treatment on the temporal profiles. Similar to our previous results (Figure 2E and S5D), we observed better segregation of treatments using LDA instead of UMAP (Figure 4B and S8E). The advantage of aggregating temporal data was even more obvious when comparing closely related conditions with varying rapamycin concentrations. Analysis of a single timepoint, either early (0.5 hours) or late (15 hours), resulted in no separation of conditions by LDA. Aggregating timepoints into cell trajectories provided much better separation, though closely related conditions still overlapped to some degree. The observed clustering based on rapamycin concentration suggests rapamycin concentration-dependent autophagy activity. This general result was also consistent with measured autophagy rates at those concentrations [12]. We were also able to identify and visualise individual features that changed as a function of rapamycin concentration (Figure 4C). These individual features were selected to represent significantly variable features in different categories (cellular, autophagy vesicle dynamics, and autophagy vesicle shape and size). This analysis shows potential in identifying autophagy response phenotypes for the same drug but at different concentrations and highlights the sensitivity of the approach.

### Image-based temporal profiling of morphological features accurately predicts the dynamic change in autophagy modulation

**Figure 5.**
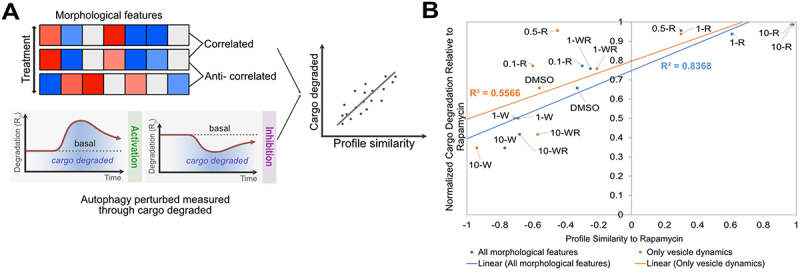
Image-based temporal profile similarity more accurately captures autophagy modulation. (A) Cartoon illustration comparing profile similarity to cargo degradation. (B) Correlation between profile similarity and normalised cargo degradation for all morphological features (blue) and autophagy vesicle numbers (Orange). This analysis is repeated by only considering individual time points in Figure S11. Abbreviations: 100 nM rapamycin (R), 1 µM Wortmannin (1-W), 10 µM Wortmannin (10-W), 1 µM Wortmannin with 100 nM rapamycin (1-WR), and 10 µM Wortmannin with 100 nM rapamycin (10-WR).

Total degradative autophagy flux provides an easy-to-interpret single measurement of autophagic capacity that can be valuable in characterising autophagy modulators for drug screening [12]. We previously demonstrated how this degradative flux can be measured directly by quantifying the cumulative rate of autolysosome turnover. However, the measurement is technically challenging due to the time series autophagy rate analysis required. We therefore assessed the predictive value of image-based temporal profiling for characterising total degradative autophagy flux without directly measuring autophagy rates using lysosomal inhibitors. We tested 9 conditions – 0.1 nM rapamycin, 0.5 nM rapamycin, 1 nM rapamycin, 10 nM rapamycin, 100 nM rapamycin, 1 µM wortmannin, 10 µM wortmannin, 1 µM wortmannin with 100 nM rapamycin, and 10 µM wortmannin with 100 nM rapamycin. These conditions previously provided a range of autophagy perturbation over 15 h [12]. We used image-based profile matching through profile correlation to assess performance because this was recently demonstrated to be a promising approach for characterising perturbations [25,26]. Image-based profile correlation was then compared to previously quantified biologically relevant changes in autophagy. Overall cargo degraded was used as the standard because it is a single measurement that captures cumulative autophagy dynamics (Figure 5A) [12].

We compared the performance of high-dimensional image-based temporal profiling to the less complex but more intuitive and commonly used measurement of autophagy vesicle dynamics (Figure S9). Relationships between treatments identified using hierarchical clustering on morphological features were distinct to those identified based solely on autophagy vesicle dynamics (Figure S10A-B). We then used profile correlation to quantitatively assess the accuracy of these measurements in capturing changes in total degradative capacity. Profile similarity correlations (Figure S10C-D) and normalised cargo degradation (Figure S10E) were analysed relative to rapamycin treatment to provide a maximum linear dynamic range with a well-characterised autophagy modulator for both measurements. We observed a better linear fit between normalised cargo degradation and profile similarity using a comprehensive set of morphological features (R^2^ = 0.8368) compared to using just autophagy vesicle dynamics (R^2^ = 0.5566) (Figure 5B). Although simple, this analysis shows the potential of using image-based temporal profiling, which includes the measurement of non-intuitive morphological features, to comprehensively evaluate autophagy perturbation and relate this to biologically relevant cellular outputs (autophagy vesicle count, amount of autophagy protein or gene expression, etc.).

We next assessed if image-based profiling on this small set of treatments could be used to predict total degradative capacity of other autophagy modulators just based on morphological features. To this end, we developed a standard curve (blue curve on Figure 5B) from a linear regression of the rapamycin and wortmannin data to predict autophagic cargo degradation for other treatments. As a test case, we chose MRT-68921, a potent inhibitor of ULK-1 (unc-51 like autophagy inhibiting kinase 1). ULK-1 plays a critical role in initiating the formation of phagophores [27]. In a nutrient-deprived environment, typical of tumour microenvironment conditions, this kinase is activated by upstream signals to meet steep metabolic demands. ULK-1 inhibition is therefore of great therapeutic interest for cancer.

**Figure 6.**
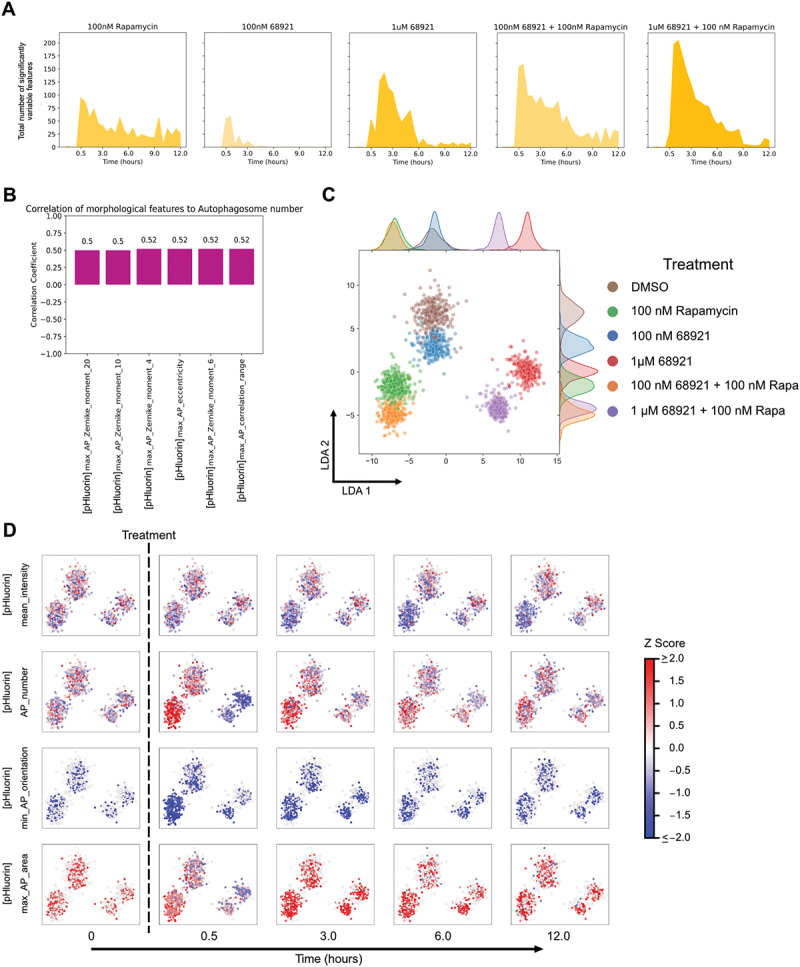
Morphological characterisation of MRT-68921 treatments and its combination with rapamycin. (A) Single cell features that varied significantly over time for different treatment concentrations. (B) Pearson correlations of the most significant features with autophagosome number, using the highest concentration of MRT-68921. (C) LDA representation of individual cells plotted using variable morphological features at all time points of treatment. (D) Changes in individual features values at different time points visualised on the LDA space.

We initially analysed the morphological properties of MRT-68921 treatment and its combination treatment with rapamycin. First, we took images over a 12 h period following treatment. We tested five conditions – 100 nM rapamycin, 100 nM MRT-68921, 1 µM MRT-68921, 100 nM MRT-68921 with 100 nM rapamycin, and 1 µM MRT-68921 with 100 nM rapamycin. Following treatment, the number of significant features increased with MRT-68921 concentration and was even higher in combination with rapamycin (Figure 6A). Of the features that varied significantly, only six were highly correlated to autophagosome number (Figure 6B), fewer than those for rapamycin treatment (Figure S8A). Visualising features on the LDA space (Figure 6C) showed that a low concentration of MRT-68921 (100 nM) had little effect, and either clustered very close to DMSO or 100 nM rapamycin. Conversely, a high concentration of MRT-68921 (1 µM) resulted in better separation from the combination treatment with rapamycin. Individual features show persistent treatment phenotypes over the 12-hour period, except for autophagosome number (Figure 6D).

**Figure 7.**
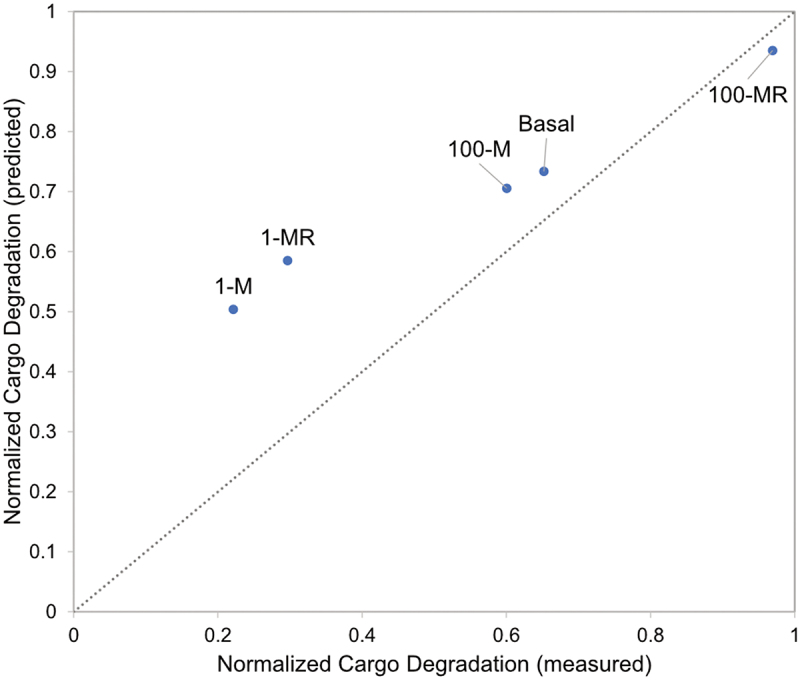
Predicted vs. measured cargo degradation for MRT-68921 treatment. Abbreviations: 100 nM rapamycin (R), 100 nM MRT-68921 (100-M), 1 µM MRT-68921 (1-M), 100 nM MRT-68921 with 100 nM rapamycin (100-MR), and 1 µM MRT-68921 with 100 nM rapamycin (1-MR).

To determine if image-based profiling had predictive value, we plotted MRT-68921 morphological profile similarity on the “standard curve” derived from rapamycin and wortmannin treatments to estimate our expected cargo degradation (Figure 5). This was compared to the measured cargo degradation using standard destructive sampling methods. Low concentrations of MRT-68921, alone and in combination with rapamycin, could be well characterised using image-based temporal profiling; however, we observed larger errors for higher concentrations of MRT-68921 (Figure 7). Thus, image-based temporal profiling holds promise in high-throughput prediction of autophagy outputs, but additional autophagy outputs beyond cargo degradation may be necessary to fully characterise the high-dimensional nature of autophagy.

One of the main predictive advantages of image-based temporal profiling is the holistic approach enables aggregating time point data since it is impossible to know *a priori* if any one timepoint in an experiment is the most predictive. To underline this point, we developed predictive models similar to the one in Figure 5B for two individual time points: early in the experiment when vesicle dynamics were most perturbed and at a late timepoint (Figure S11A). Aggregated timepoints performed better than the early timepoint. Suprisingly, aggregated timepoints performed just as well compared to the late timepoint based on their overlapping 95% confidence intervals (Figure S11B). However, since it is impossible to anticipate which timepoint would provide the best predictive value, a holistic approach is safer. Aggregating time points can also reliably holistically account for changes over time rather than through individual snapshots that may remove valuable context (the same autophagic state could be reached from opposite directions, for example).

## Discussion

Measuring autophagy is challenging due to its dynamic and multistep nature. Established methods can accurately measure each autophagy step in the form of rates with high sensitivity, but rely on destructive sampling that limits scalability [12,14]. High-throughput characterisation of autophagy would enable accelerated drug screening and a better understanding of the underlying mechanisms. Even so, existing high-throughput methods involve destructive sampling, which limits the ability to associate a phenotype with the temporal change in the autophagy state at the single cell level [15,23]. For example, the governing features of autophagy-dependent cell death are not fully understood. Monitoring various autophagy rates over time to derive a correlation between autophagy and cell death is not possible using existing methods since the same cell cannot be followed to the point of cell death using these approaches. Image-based temporal profiling offers a comprehensive and high-throughput approach for characterising cellular phenotypes and perturbations with single-cell trajectories [28,29]. We performed a proof-of-concept study to evaluate the potential of using image-based profiling for characterising autophagy modulation.

We developed a random forest classifier with an accuracy of ~90% to differentiate rapamycin, wortmannin, and DMSO treatment. To interpret the importance of individual features in the classification, we used the SHAP feature importance method. Our findings demonstrate that using SHAP to interpret machine learning models can assist in the identification of governing features and biologically relevant phenotypes from large datasets. While we used a model with satisfactory performance in identifying differentiating features between the three treatments, we acknowledge that the classifier accuracy could be further improved by optimising hyperparameters or using other classification algorithms.

For this proof-of-concept study we focused on characterising morphological phenotypes at a bulk level and did not fully leverage the benefits of the single cell temporal resolution. We observed heterogeneity in multiple features and time points for both rapamycin and wortmannin treatments. For example, wortmannin treatment caused a small portion of cells to have the opposite response compared to the rest for min_AP_contrast_mean and max_AP_area features at 30 min post treatment. Identifying features correlated to single-cell heterogeneity could reveal novel insights. Coupling this kind of analysis with additional autophagy-related biosensors could also elucidate biological mechanisms involved in heterogeneity [30,31].

We investigated morphological features that closely correlate with autophagosome number as potential alternative biomarkers for autophagy. This analysis strategy may be useful when performing perturbation experiments in cells that produce or maintain a low number of autophagy vesicles and are therefore not easily tracked. When combined with machine learning models, could serve as a bridge to link biologically nontrivial morphological features to established measures of autophagy in systems where these classic measurements are challenging.

Image-based profile correlation between a standard reference drug (rapamycin in this study) and other treatments showed that overall effects on autophagy flux and the degradative capacity of the pathway can be captured to some degree using this high-dimensional, non-destructive technique. For example, the correlation between rapamycin and DMSO profiles could be used as a threshold and any treatment with a higher correlation would represent an autophagy inducer or vice-versa. Similarly, to identify autophagosome fusion modulators or autolysosome degradation modulators, additional reference drugs that affect those specific steps will be needed for comparison. However, it is worth noting that there could be pathway-specific morphological variations between modulators that affect autophagy similarly. For instance, while rapamycin and another treatment may induce autophagy, they may not necessarily affect the same morphological features. Profiling morphological changes induced by a wider range of perturbations would aid in identification of pathway-specific morphological alterations and features that change globally in response to perturbations with a similar effect on autophagy. This could also reveal how distinct mechanisms lead to the same cellular output.

We used a simple profile similarity approach over more complex machine learning models to avoid overfitting with a limited amount of data. Machine learning models require richer training data and identification of features that are universally correlated with a general class of perturbations to achieve accurate prediction. For instance, PI3K inhibitors and ULK1 inhibitors both inhibit early autophagy events, but might have different effects on the morphology. Therefore, a model trained on PI3K inhibitors may not accurately predict ULK1 inhibitors. Unlike machine learning, a profile similarity approach does not rely on pre-weighted features, which allows capturing the diverse differences in morphology. Nevertheless, machine learning models could be incredibly useful to predict autophagy states. Training a model at various concentrations could be used to predict how changes in autophagy impact more complex cellular decisions, such as cell death, at a single cell level.

This approach also made use of temporal data through the aggregation of features at all time points, as we hypothesise that using and aggregating temporal data is an advantage. Visualising aggregate versus snapshot data shows us that the embedding of the aggregated timepoint data is able to capture subtle differences across timepoints. It also supports a systematic and unbiased approach in determining the predictivity of a model, since it is very difficult to anticipate which single time points are the most impactful to make predictions from. In principle, the concepts applied here are not unique to autophagy. Thus, image-based temporal profiling and holistic integration of dynamic data could be applied to pathways orthogonal to autophagy for which biosensors exist. It will also be important to test how much temporal resolution is required. Testing the performance of image-based profiling on subsets of temporal data, as opposed to single snapshots in time, could help determine if one can take advantage of the holistic dynamic analysis without increasing data requirements substantially.

We also acknowledge several nuances are of this automated image analysis. First and foremost, there can be systematic over- or under-counting of features. For a blinded and automated analysis pipeline, a systematic bias can be accounted for through the use of appropriate controls. We addressed this through the use of well-characterised autophagy modulating chemicals for which specific feature dynamics had already been established. If over- or under-counting occurs in a treatment-specific manner, this would introduce a non-uniform bias that may not be well controlled. Thus, careful manual inspection of the automated pipeline under different conditions is critical. Moreover, for poorly performing conditions (such as high concentrations of MRT 68921, Figure 7) could be targeted for extra manual inspection to identify potential condition-specific biases.

Future opportunities lie in comparing performance between pathway-specific and pathway-agnostic phenotypic characterisation for drug screening. The autophagy-related phenotypic characterisation performed in this study is a pathway-specific phenotypic characterisation approach, while assays such as Cell Painting are pathway-agnostic [32]. Assays like Cell Painting offer an unbiased and holistic measurement of cellular phenotypes. Thus, Cell Painting has the potential to capture off-target effects, which may be overlooked if a specific pathway is monitored and targeted alone. Conversely, Cell Painting is limited by the temporal resolution it can achieve, which might be important for highly dynamic biological processes, such as autophagy. Therefore, studies comparing the performance of both methods would be valuable in determining the optimal approach for characterising perturbations. Additionally, if both live cell imaging and Cell Painting provide complementary data, both approaches can be combined for a comprehensive autophagy state characterisation. In conclusion, image-based temporal profiling of autophagy is an exciting approach that can be applied in various contexts to improve fundamental understanding of the autophagy pathway as well as expedite drug screening for various disease indications.

## Materials and methods

### Cell culture, chemical treatments, and live cell imaging

Cell culture, generation of the A549- and U2OS-pHluorin-mKate2-LC3 reporter cell lines, image acquisition for live cell imaging, and chemical treatments using rapamycin (Selleck Chemicals, S1039), wortmannin (Selleck Chemicals, S2758), and DMSO (Sigma Aldrich, 472301) were previously discussed [12]. MRT68921 (Selleck Chemicals, S7949) was also used for treating cells with the same procedure. At least three individual replicates were performed for collecting data for rapamycin, wortmannin, and MRT-68921 experiments (Table S2). The third replicate consisted of technical duplicates. Any disturbed images were manually identified, then excluded from analysis through the automated image analysis pipeline. Disturbed images consisted of those where cells moved out of the focal plane during imaging, therefore rendering the images unusable. The number of cells/images analysed and disturbed images removed for each condition is listed in Table S2.

### Image processing, cell and puncta mask generation, and cell tracking

Microscope images taken with the Nikon Ti2 Eclipse were acquired using NIS Elements 5.11.01 and produced a “.nd2” format file containing all the images and their associated metadata. Puncta masks for autophagosomes and autolysosomes were created using the NIS Elements spot detection tool. Processing and analysis were performed on GFP (pHluorin) and TRITC (mKate2) channel images. To improve spot detection, the images were background corrected through the rolling ball method using a radius of 0.98 µm. The spot detection tool was programmed to detect circular areas approximately 0.8 µm in size with a contrast value of 5 for GFP images, and 6 for TRITC images. To capture puncta of different sizes, the Grow Bright Regions to Intensity operation was used. To determine which puncta were autophagosomes, puncta that were overlapping in both the TRITC and GFP regions, denoted by the TRITC HAVING (TRITC AND GFP) binary operation named “BOTH”. For the detection of autolysosomes, the remaining puncta in the TRITC channel not detected by BOTH were detected using the binary operation TRITC SUB BOTH. The general analysis file is available on GitHub. Examples of spot detection for each treatment is shown in Figure S1. Automated Spot detection can result in over- or under-detection of autophagosomes. However, this is a systematic and blinded bias that can be accounted for through the use of appropriate negative controls.

Following the initial analysis in NIS elements, all subsequent analysis was performed in Python. All code is available on Github. Cell masks were generated using the Cellpose algorithm [33]. A model was trained to improve cell masking accuracy by manual input of cell mask boundaries on an image [34]. Cellpose then learns from these new masks and applies it to a different image contained in the same directory. This process was repeated until cell masking accuracy was confirmed satisfactory by manual inspection. The total cell count detected using cell masks was also compared to the cell count based on nucleus count Hoechst staining in separate experiments.

To obtain accurate single-cell temporal data, the bTrack algorithm [35] was used to track individual cells across a sequence of images that were captured using a microscope. The algorithm uses cell masks and custom-optimised parameters (available on GitHub) to generate cell tracks. Accuracy of cell tracking was confirmed manually for various independent experiments. An example track series can be found on GitHub. After the division of a cell, one of the daughter cells continues to have a parent ID and these cells were followed for the entire time course. The other daughter’s cell information is discarded. This leads to the loss of immense data and could be improved in the future. Cell masks, puncta masks, and cell tracks are required before proceeding to feature extraction.

### Feature extraction, data preprocessing, standardisation, and interpretation

Morphological features were extracted for three biological categories: Whole cell, Autophagosome (AP), and Autolysosome (AL). GFP channel images were used for extracting autophagosome and whole cell features, while TRITC channel images were used for autolysosome features. GFP channel images have a better signal to noise ratio compared to TRITC, hence they were used for extracting whole cell features. Regionprops from skimage.measure was used for extracting some of the features [36]. The mean and range of all 14 haralick texture features calculated in all directions were included as features. 25 Zernike moments were calculated using 0.5*major_axis_length of the object as the radius. Haralick features and Zernike moments were estimated using Mahotas package [37]. After calculating features for each vesicle, descriptive statistics of each feature for all the vesicles in an individual cell were calculated. The descriptive statistics include mean, median (50%), lower quartile (25%), upper quartile (75%), maximum value (max), and minimum value (min). Autophagosome and autolysosome features were prefixed with AP and AL respectively and were further prefixed with descriptive statistics. A list of 949 features extracted along with their respective categorisation as biological entity (cells, autophagosomes, or autolysosomes) and morphological type (shape, intensity, texture) for each feature is available on GitHub.

For data organisation purposes, each image file was systematically named to include the replicate date, well name, well position, and the channel used to take the image. This made it possible to link the well name and position to its corresponding treatment for standardisation. This also ensured that experiments performed on different days did not have duplicate labels.

Features containing NAN values were removed. Each individual feature for each cell was centralised using the median value of DMSO-treated cells (median (X_DMSO_)) and divided by 1.2532 times the mean absolute deviation (MAD(X_DMSO_)) of that feature from the respective plate (equation is shown below). 1.2532 times the mean absolute deviation (MAD (X_DMSO_)) is approximately equal to the standard deviation [38]. This standardised data was referred to as modified Z score. This approach was used to account for the batch effects between experiments.

For visualising single-cell features over time, cluster maps were generated using the clustermap function in the seaborn package [39]. These were generated for features that changed significantly after treatment as well as for features that counted the number of autophagy vesicles.

### Feature selection

All features with a median modified Z score ≥ 0.5 with an adjusted P-value < 0.05 were considered significant. Mann-Whitney U statistical test was used for estimating the statistical significance of each feature between DMSO-treated and small molecule treated cells. Benjamin–Hochberg method was used for false discovery rate correction.

### Flow cytometry

Cells were harvested and resuspended in PBS+ 1% FBS after treatment with DMSO and 100 nM rapamycin for the indicated time. The mean FITC intensity of the cells was analysed using 488 nm laser on CytoFLEX S Flow cytometer. A minimum of 5,000 events were analysed for each of the three independent replicates performed. Gating was applied to exclude 99.9% of non-fluorescent base strain A549 cells.

### Dimensionality reduction

2D UMAPs were generated using UMAP package on Python [40]. Hyperparameters used for generating the maps were neighbor = 250 and mindist = 0.90. LDA plots were generated using the sklearn.discriminant_analysis package using default parameters.

### Random forest classification and feature importance

Features that varied significantly after treatment at all time points were combined at a single cell level. Features that were correlated with a Pearson correlation of 0.75 and above were removed except the first feature to remove redundancy. A lower correlation threshold of 0.75 was used to eliminate correlated features as co-linearity can considerably affect the feature importance interpretation [41]. RandomForestClassifier algorithm from sklearn.ensemble was used as the model. Random forest model accuracy was measured using a 5-fold cross-validation method. To elaborate, data was split into 5 folds, where 4 folds were used for training while the remaining fold was used for testing the accuracy of the prediction. We iteratively performed this step 5 times and calculated the average and standard deviation of the micro F_1_ score. One thousand trees were used as the random forest model, entropy as the criterion, and other hyperparameters are left as default. F_1_ micro score package from sklearn.metrics was used for calculating the F_1_ score [21]. A minimum of 70 cells were used for testing the accuracy of the model. Shapley Additive exPlanations (SHAP) method was used to interpret the feature importance from the classifier [22]. In short, SHAP assigns feature importance value for each feature by considering all possible combinations of features and the contribution of an individual feature to the final prediction. This was employed using SHAP package in Python.

### Hierarchical clustering and Pearson correlation

All features that varied significantly from DMSO-treated condition for all treatments at all time points were aggregated at a single cell level. In parallel, we used just the number of the autophagosome, and autolysosome features from all time points for comparison. Hierarchical clustering was performed based on median profiles of each treatment. clustermap function from seaborn package was used for clustering. “*average”* method and “*euclidean”* metric was used for generating the clusters. Pearson correlation was calculated using the median profiles of each condition.

### Statistical analysis

Statistical tests used for determining significance are mentioned in the corresponding figure legend. The feature selection section describes statistical tests used for determining significantly variable features.

The 95% confidence interval for each model was calculated using scipy.stats.

## Supplementary Material

Revised Supplemental Information.docx

## Data Availability

Raw feature data (Tables S3-14) and preprocessed data (Tables S15-20) is available on https://figshare.com/articles/dataset/Supplemental_Data/25697670 Due to the large size of raw datasets, raw image data is available upon request. All original code for feature extraction, preprocessing, and analysis are available on https://github.com/shahlab247/ATG_morphological_profiling. Any additional information required to reanalyze the data reported in this paper is available on request.

## Abbreviations

MAP1LC3/LC3: microtubule-associated protein 1 light chain; AP: Autophagosomes; AL: Autolysosomes; SHAP: Shapley Additive exPlanations; DMSO: Dimethyl Sulfoxide
