# Supplementary material for: Image-based temporal profiling of autophagy-related phenotypes
Source: Autophagy Rep. Author manuscript; Available in PMC 2025 Apr 25. (PMC11988254; doi:10.1080/27694127.2025.2484835)
Supplement: Supplemental Information [file NIHMS2070057-supplement-Supplemental_Information.docx]

**Supplemental Information**


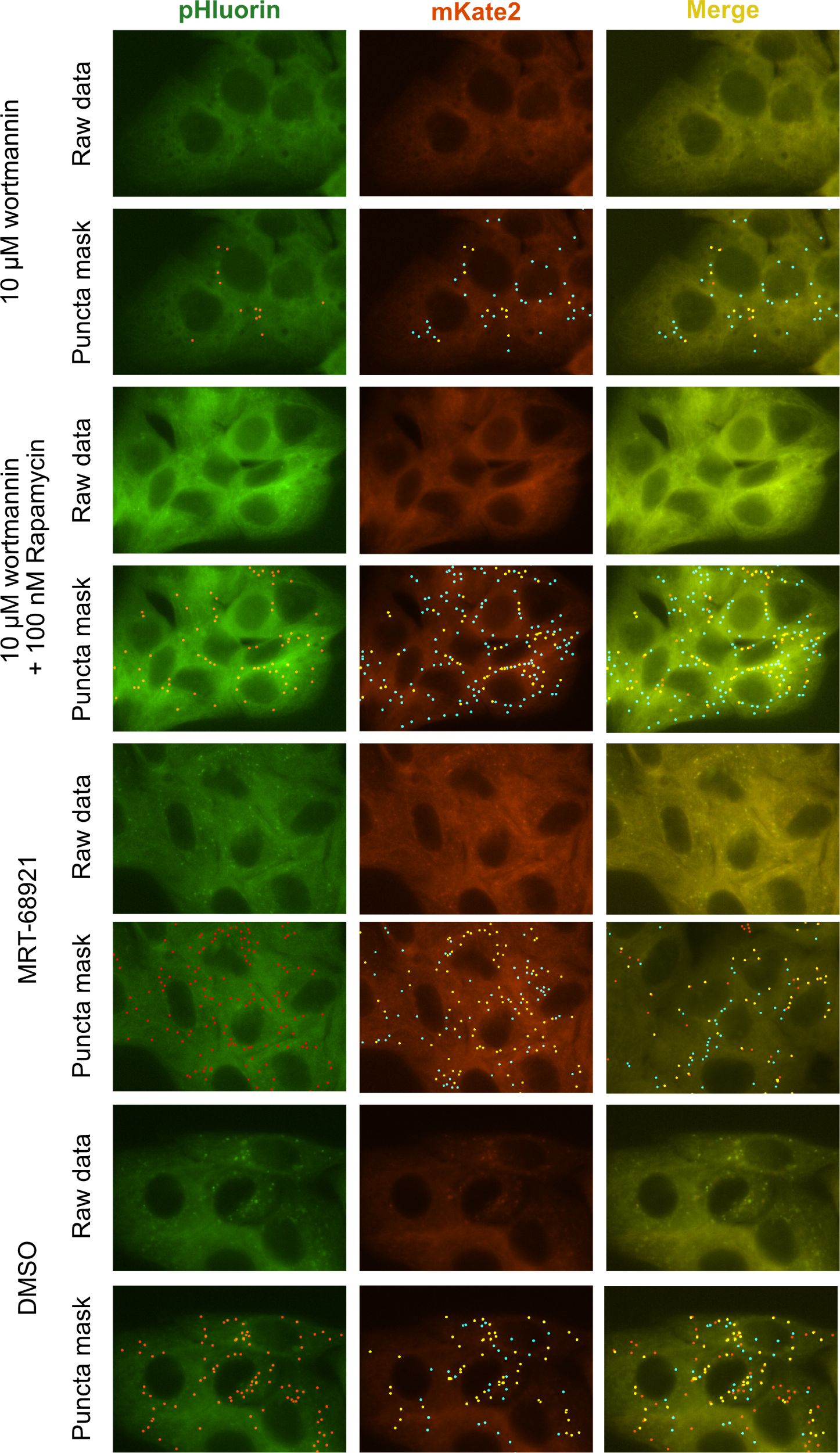


**Figure S1**. Sample representative microscopy images of cells treated with wortmannin, a combination of wortmannin and rapamycin, DMSO, and MRT-68921 along with sample images of puncta masks generated by NIS Elements. The same method is applied to puncta masks regardless of treatment, thus equally applying any systematic bias inherent to the detection method. Red puncta are pHluorin-positive. Yellow puncta are pHluorin- and mKate2-positive and represent autophagosomes. Cyan puncta are mKate2-positive only and represent autolysosomes.


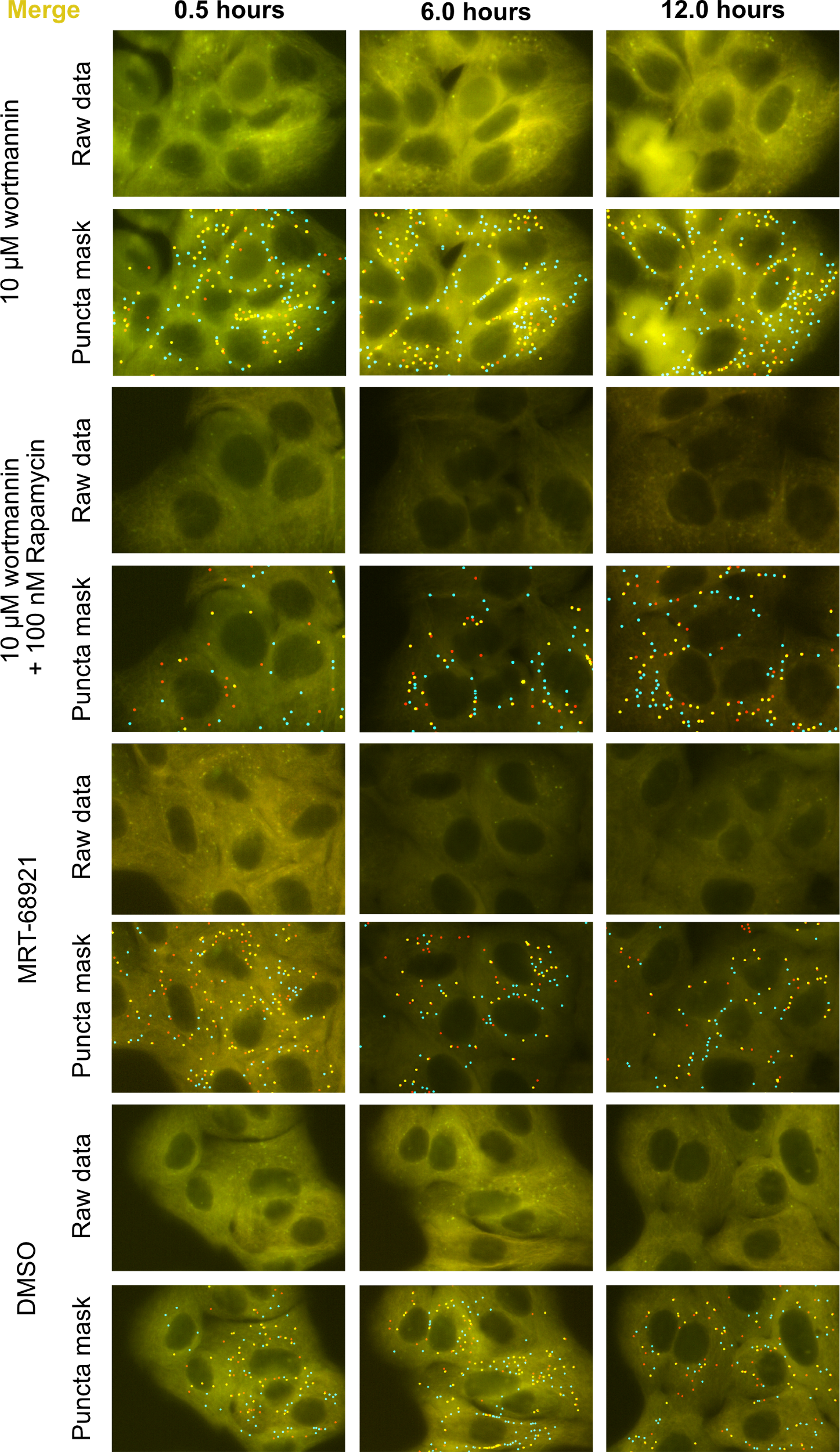


**Figure S2**. Sample representative microscopy images of cells treated with wortmannin, a combination of wortmannin and rapamycin, DMSO, and MRT-68921 along with sample images of puncta masks generated by NIS Elements at 30 minutes, 6 hours, and 12 hours after treatment. The same method is applied to puncta masks regardless of treatment, thus equally applying any systematic bias inherent to the detection method. Red puncta are pHluorin-positive. Yellow puncta are pHluorin- and mKate2-positive and represent autophagosomes. Cyan puncta are mKate2-positive only and represent autolysosomes.

**
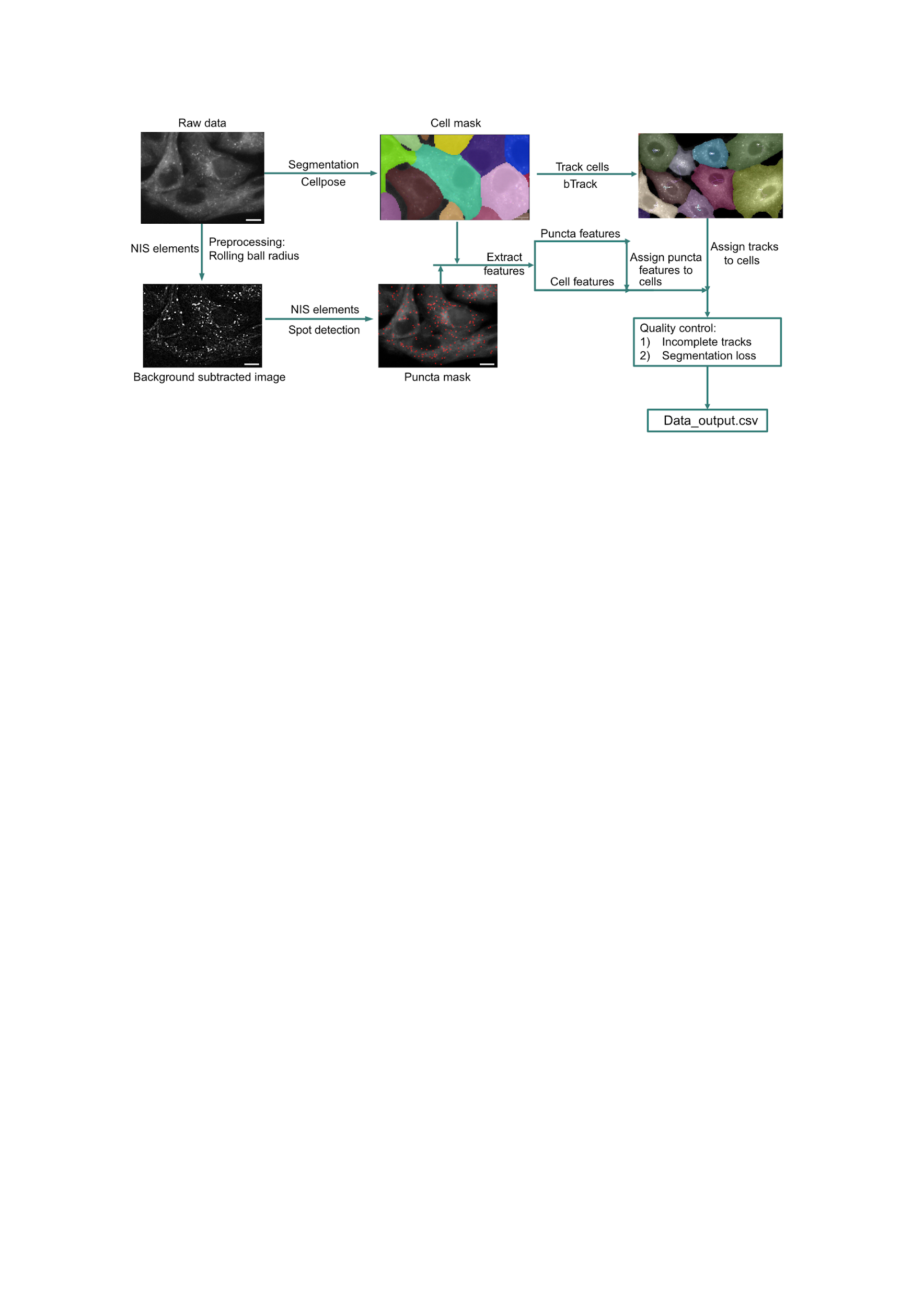
**

**Figure S3**. Schematic of the image analysis pipeline.


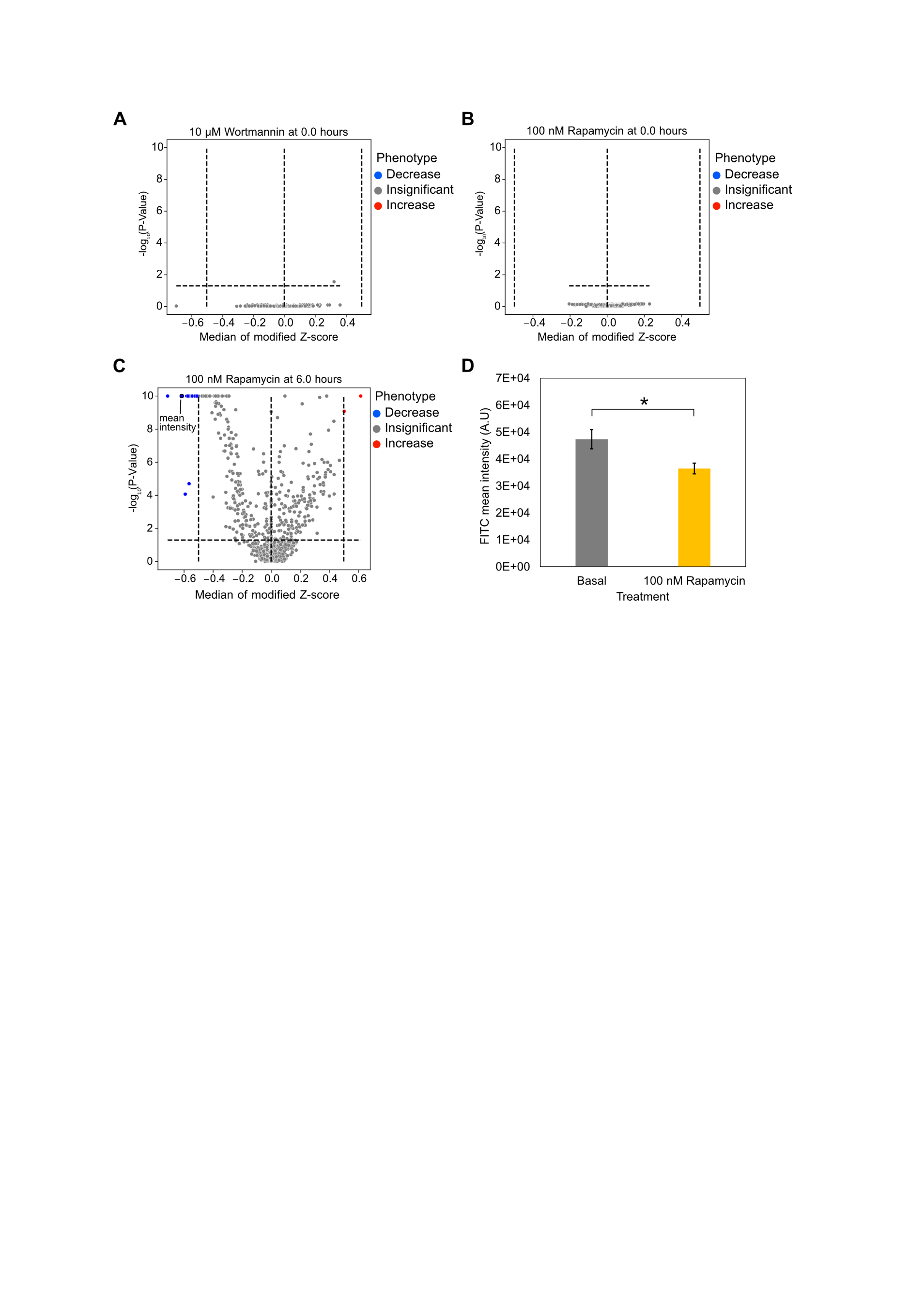


**Figure S4**. Image analysis pipeline validation. (**A-B**) Volcano plots of cellular features before treatment. (**C**) Decrease in cellular mean intensity (pHluorin channel) measured using image analysis pipeline after treating with 100 nM rapamycin for 6 h. (**D**) Decrease in cellular mean intensity (pHluorin/FITC) measured using flow cytometry after treating with 100 nM rapamycin for 6 h. Three independent replicates were performed. (*) represents p-value < 0.05. An independent two-tailed t-test was used to calculate the P-value.


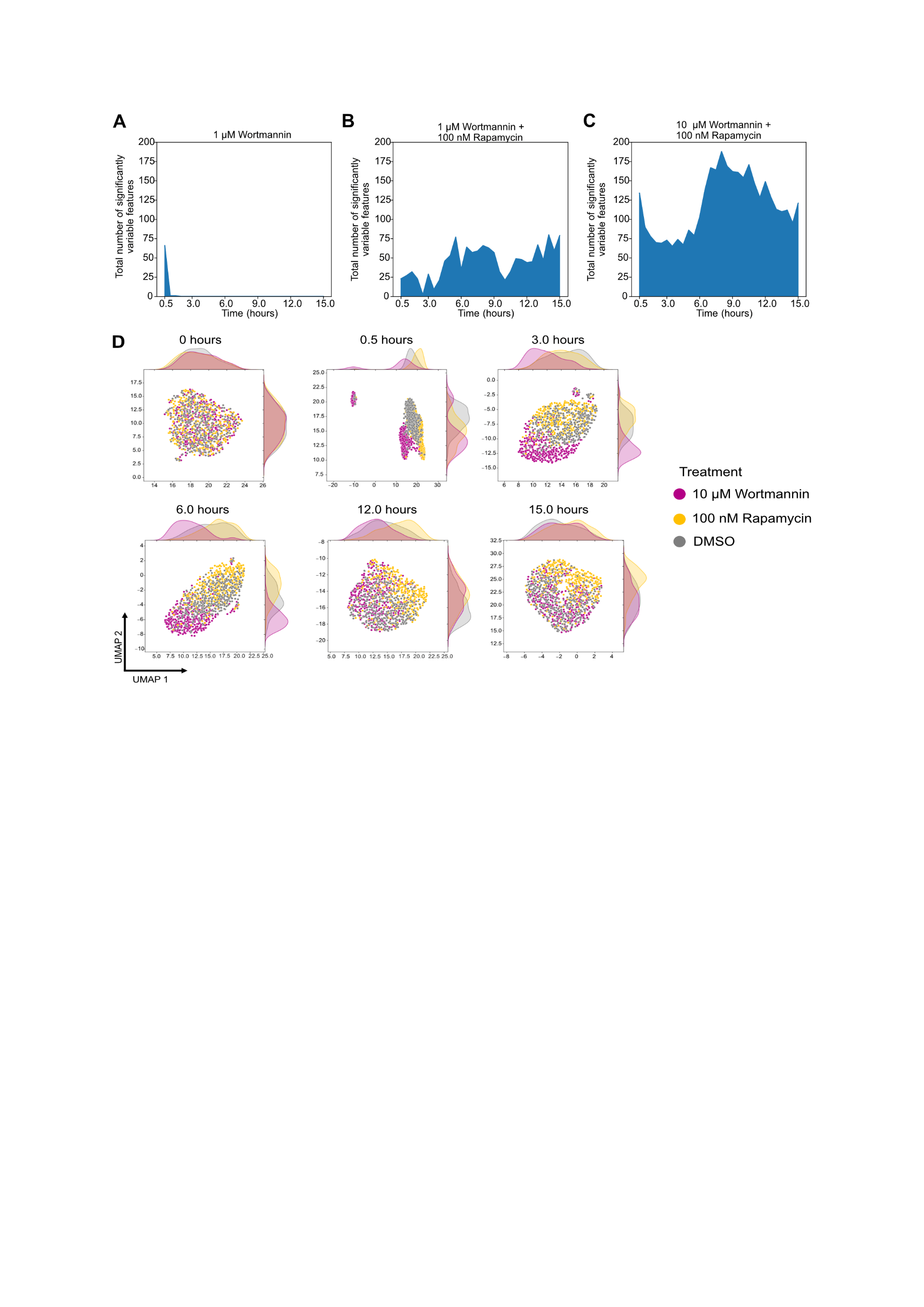


**Figure S5**. Concentration dependent effect of wortmannin on dynamic morphological features. Change in number of significantly variable features as a function of time after treating with (**A**) 1 µM wortmannin (**B**) 1 µM wortmannin and 100 nM rapamycin, and (**C**) 10 µM wortmannin and 100 nM rapamycin. (**D**) UMAP of cells treated with DMSO, 10 µM wortmannin, and 100 nM rapamycin at different time points. A minimum of 300 cells were analyzed for each condition. Features that varied significantly with a median modified Z-score > 0.5 at specific time points were used for generating the UMAPs. For 0 h, features from 0.5 h were used.


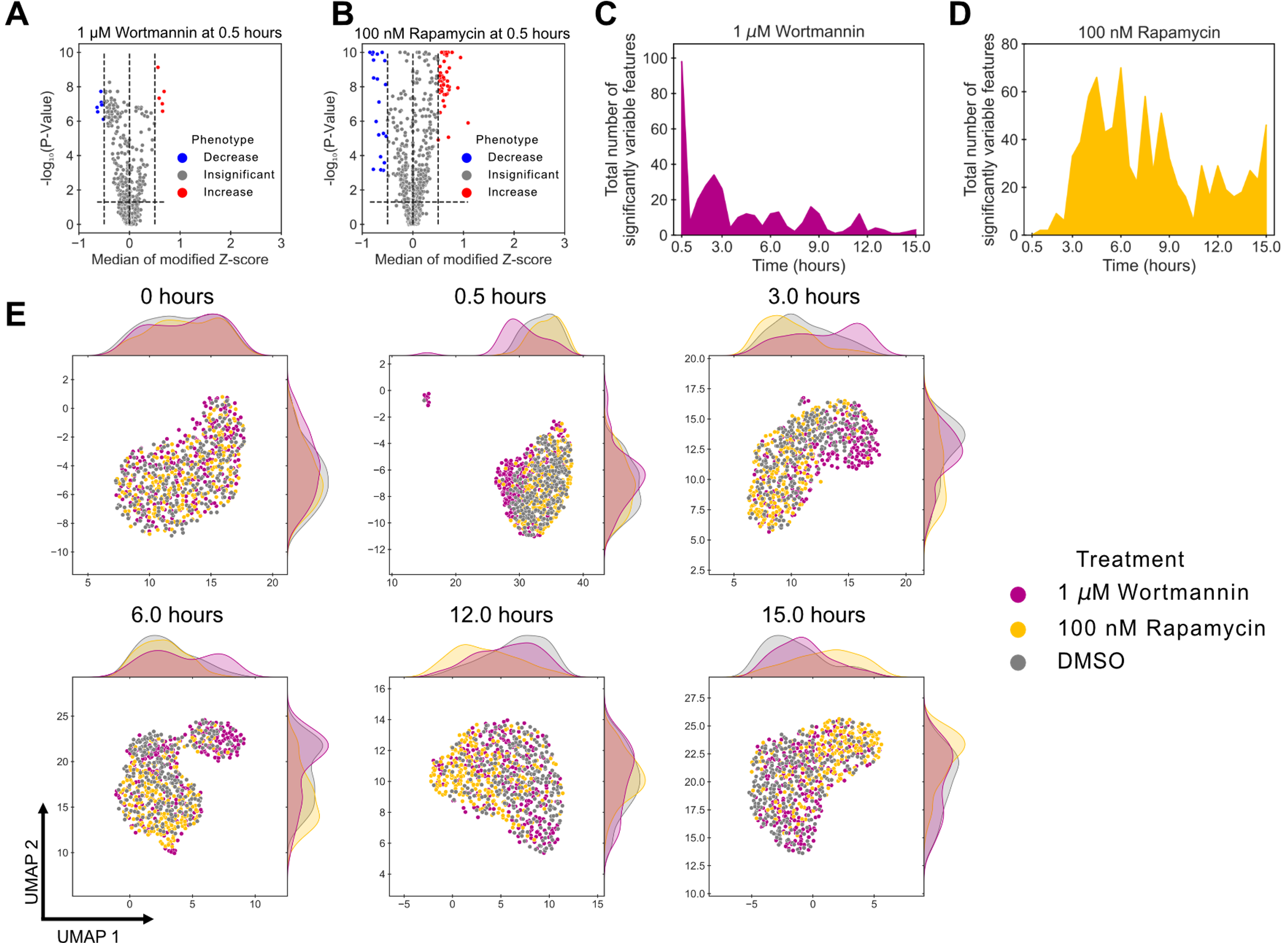


**Figure S6.** Temporal change in morphological features after rapamycin and wortmannin treatment in U2OS cells. (**A-B**) Volcano plots of cellular features after 30 min of treatment with (**A**) 1 µM wortmannin and (**B**) 100 nM rapamycin, respectively. (**C-D**) Cellular features that varied significantly as a function of time for 1 µM wortmannin and 100 nM rapamycin, respectively (**E**) UMAP of cells treated with DMSO, 1 µM wortmannin, and 100 nM rapamycin at different time points. A minimum of 300 cells were analyzed for each condition. Features that varied significantly with a median modified Z-score > 0.5 at specific time points were used for generating the UMAPs. For 0 h, features from 0.5 h were used.


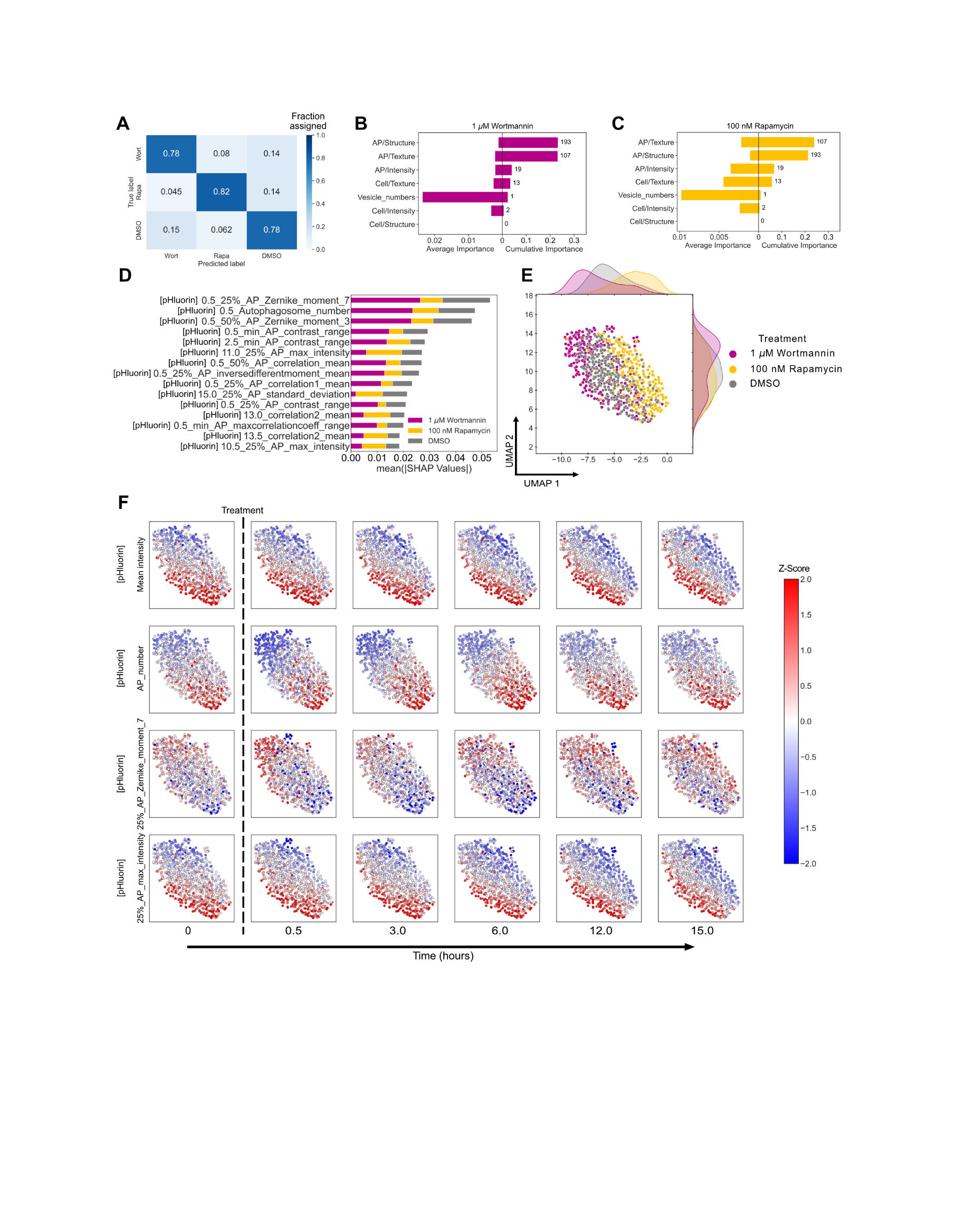


**Figure S7**. Differentiating rapamycin and wortmannin treatments and governing features in U2OS cells aggregated over all timepoints. (**A**) A confusion matrix to visualize the performance of the random forest model in classifying 1 µM wortmannin and 100 nM rapamycin and DMSO treatments. A minimum of 70 cells for each condition were used for testing the accuracy of the model. (**B-C**) Cumulative and average importance of feature categories in classifying wortmannin and rapamycin treatments, respectively. (**D**) The top 15 features with the highest mean absolute SHAP values. (**E**) UMAP of individual cells constructed using variable features from all time points. A minimum of 300 cells were analyzed for each condition. (**F**) Change in feature values at a single cell level with time after treatment with wortmannin and rapamycin.


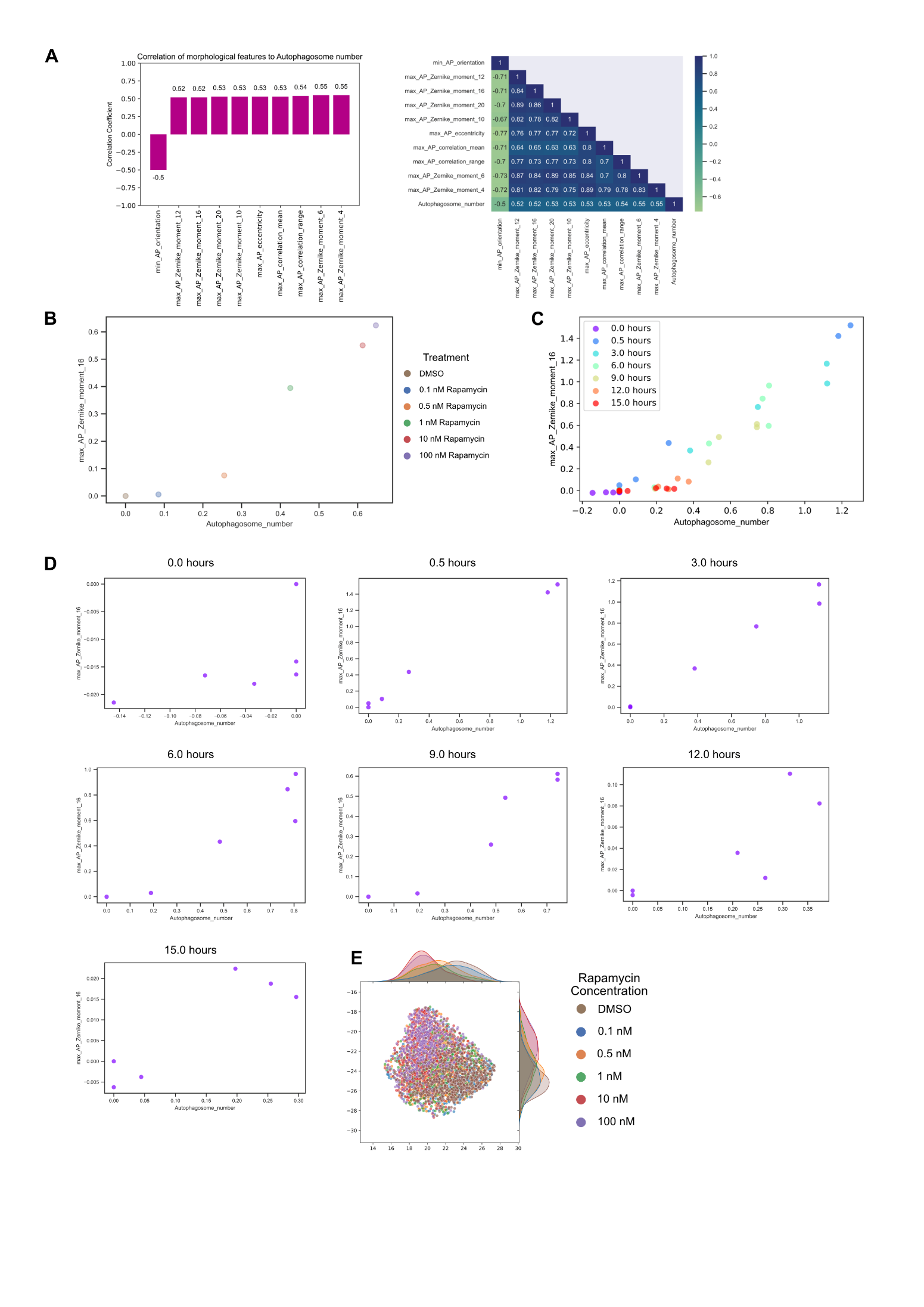


**Figure S8.** Morphological features that are highly correlated with autophagosome number to identify alternative autophagy biomarkers. (**A**) Pearson correlation between features that are highly correlated (>0.5) to autophagosome number based on all rapamycin concentrations. Those features are correlated to one another as well. (**B**) Concentration-dependent behavior of max_AP_Zernike_moment_16 relative to Autophagosome_number. Median modified Z-score values of max_AP_Zernike_moment_16 and Autophagosome_number at different concentrations of rapamycin at all time points. (**C**) Time trajectory behavior of both features at all concentrations. Median modified Z-score values of max_AP_Zernike_moment_16 and Autophagosome_number at different time points. Each point of the same color corresponds to a different rapamycin concentration for that time point. (**D**) Time trajectory behavior in (**C**) decomposed into individual time points. (**E**) UMAP representation of individual cells at different rapamycin concentrations.


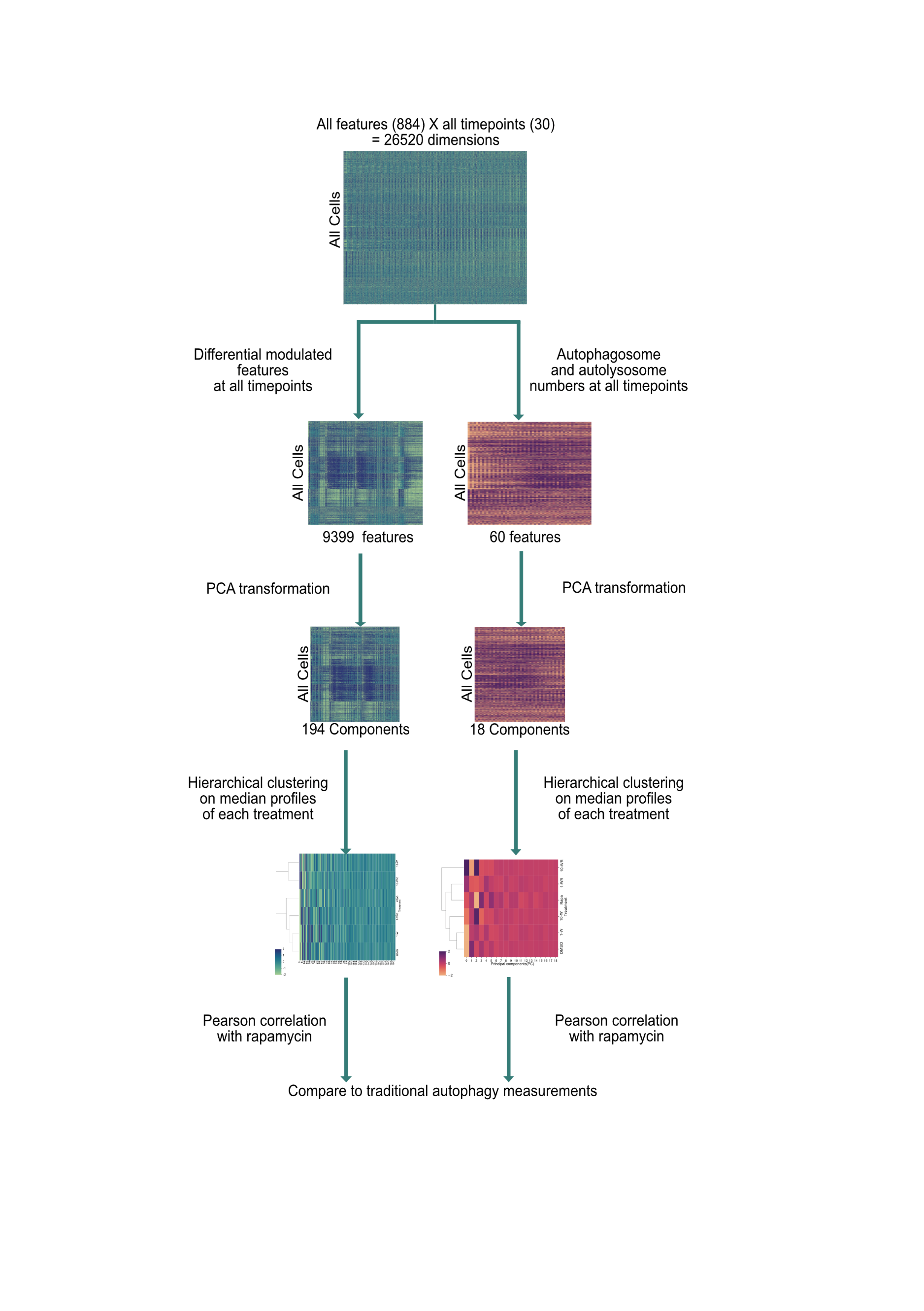


**Figure S9**. Stepwise procedure for calculating profile correlation. PCA was performed on features to reduce redundancy. 90% of the variance is retained after PCA.


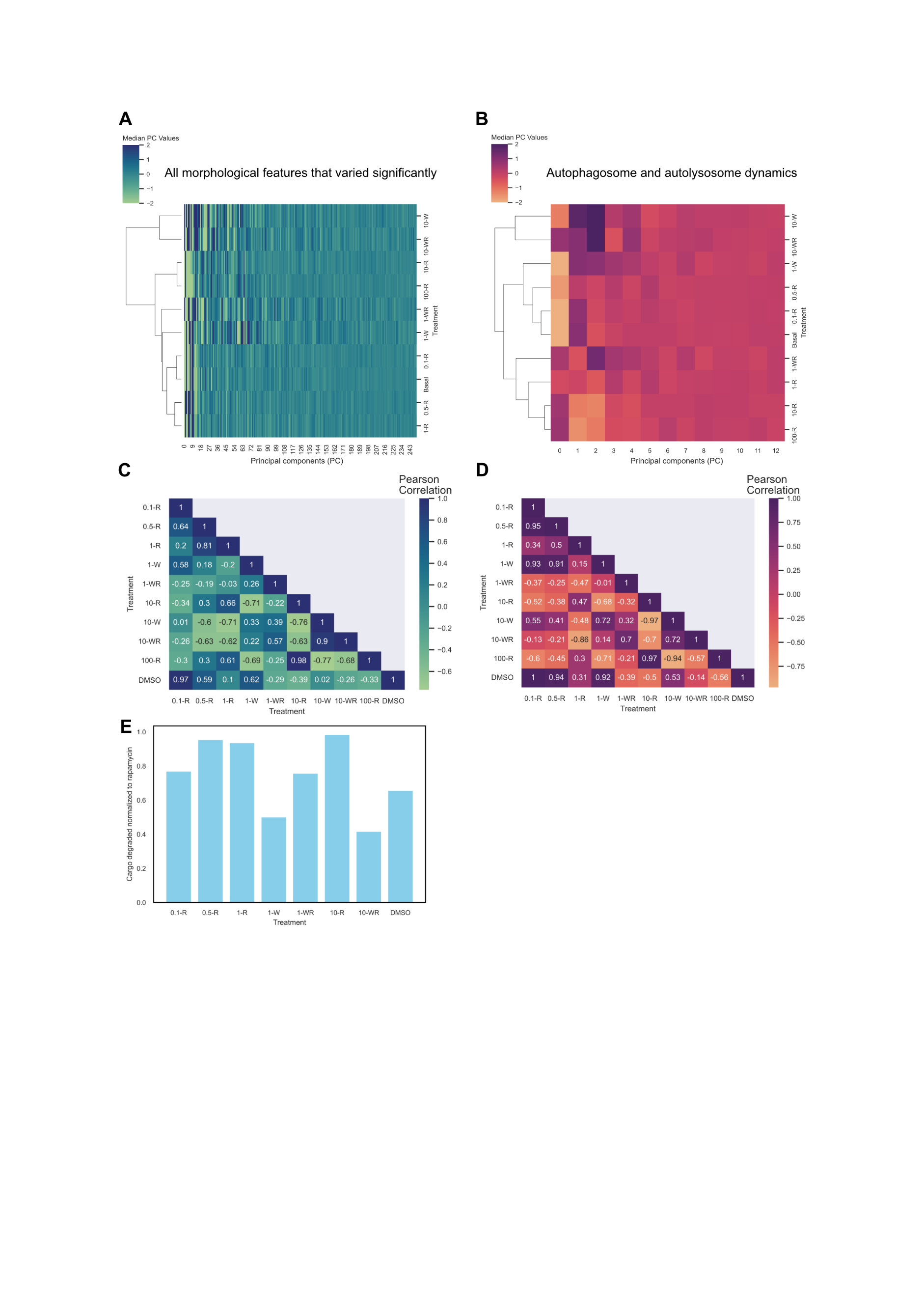


**Figure S10**. Assessing similarities among treatments using morphological feature and autophagy vesicle number dynamics. (**A**) Hierarchical clustering of median profiles after principal components analysis (PCA) based on temporal image features. The color bar for heatmap represents median principal component (PC) values. (**B**) Hierarchical clustering based on median profiles after PCA based on just temporal vesicle numbers. The color bar for heatmap represents median principal component (PC) values. (**C**) Pearson correlation between profiles based on temporal morphological features. (**D**) Pearson correlation between profiles based on just temporal vesicle numbers. (**E**) Cargo degradation normalized to rapamycin treatment. Figure adapted from previous publication [1]. Abbreviations: 10 µM wortmannin (10-W), 1 µM wortmannin (1-W), 0.1 nM rapamycin (0.1-R), 0.5 nM rapamycin (0.5-R), 1 nM rapamycin (1-R), 10 nM rapamycin (10-R), 100 nM rapamycin (100-R), 10 µM wortmannin with rapamycin (10-WR), and 1 µM wortmannin with rapamycin (1-WR).


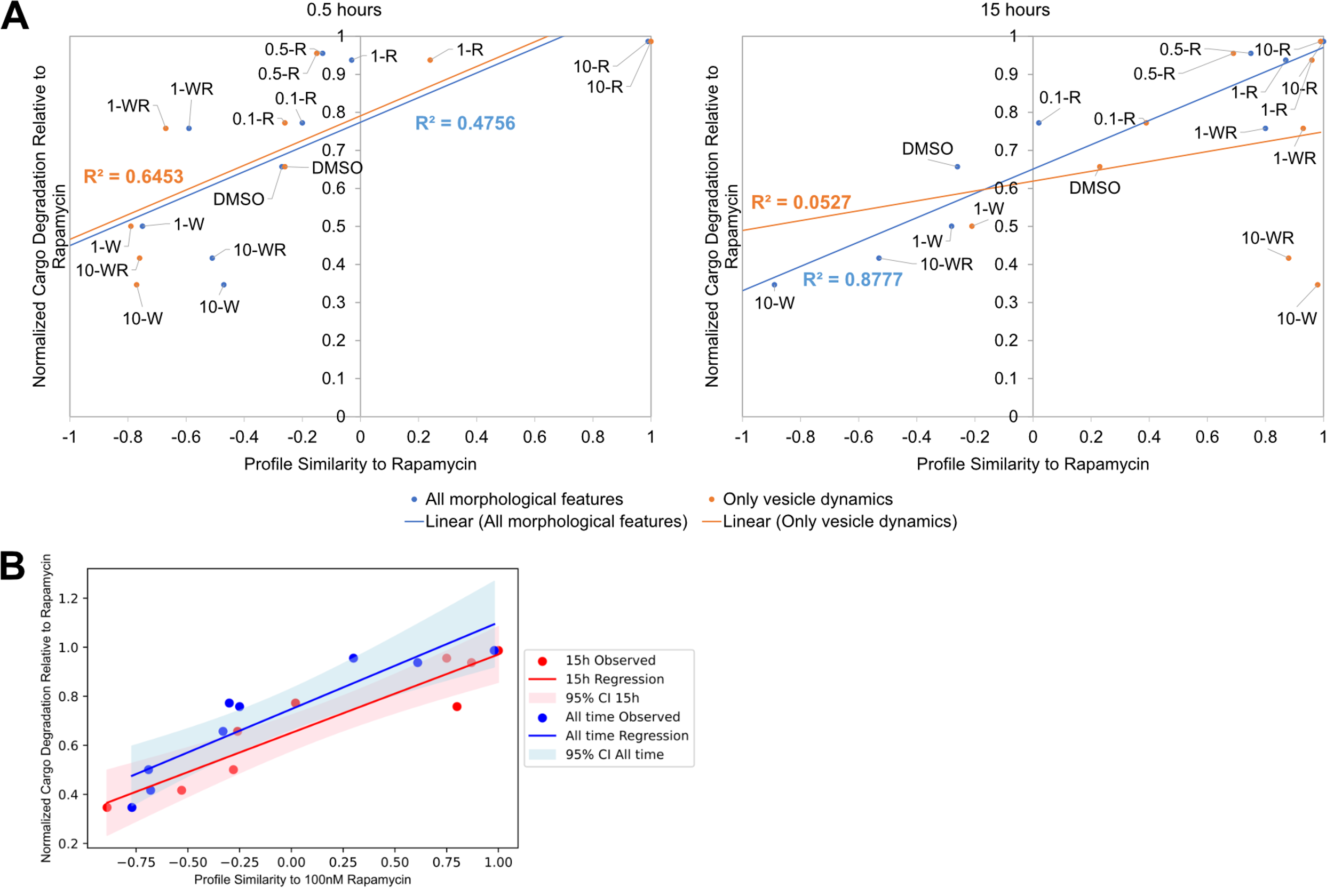


**Figure S11.** Comparison of using individual vs. aggregate time points (as done in **Fig 5**). (**A**) (Left) Correlation between profile similarity and normalized cargo degradation for all morphological features (blue) and autophagy vesicle numbers (orange) by only looking at images 0.5 hours after treatment. (Right)Correlation between profile similarity and normalized cargo degradation for all morphological features (blue) and autophagy vesicle numbers (orange) by only looking at images 15 hours after treatment. (**B**) Comparison of the 95% confidence intervals of **Fig 5B** and **Fig S10B** using morphological features. Predictive potential of the two models have considerable overlap. Abbreviations: 10 µM wortmannin (10-W), 1 µM wortmannin (1-W), 0.1 nM rapamycin (0.1-R), 0.5 nM rapamycin (0.5-R), 1 nM rapamycin (1-R), 10 nM rapamycin (10-R), 100 nM rapamycin (100-R), 10 µM wortmannin with rapamycin (10-WR), and 1 µM wortmannin with rapamycin (1-WR).

| Feature |
| --- |
| 12.0_mean_intensity |
| 12.0_min_intensity |
| 12.0_mean_AP_max_intensity |
| 12.0_25%_AP_max_intensity |
| 12.0_25%_AP_sumaverage_mean |
| 12.0_25%_AP_sumvariance_mean |
| 12.0_25%_AP_variance_mean |
| 12.0_50%_AP_max_intensity |
| 12.0_75%_AP_max_intensity |
| 12.0_25%_AP_contrast_mean |

**Table S1**. List of the top 10 most significantly variable features after drug treatment. Feature information includes the time point, the descriptive statistic, the biological entity (cell, AP, AL), and the feature being quantified.

| Experiment | Replicates | Images Analyzed | Total Images | Disturbed Images | Conditions | Cells Analyzed |
| --- | --- | --- | --- | --- | --- | --- |
| wort_three_reps_zscored_with_two_reps_12202022 | 3 | 3360 | 6020 | 2660 | Basal | 384 |
|  |  |  |  |  | 1W | 336 |
|  |  |  |  |  | 10W | 347 |
|  |  |  |  |  | 1WR | 416 |
|  |  |  |  |  | 10WR | 332 |
|  |  |  |  |  | 100R | 369 |
|  |  |  |  |  | **Total** | **2184** |
| data_with_p_values_rapa_conc | 4 | 4200 | 5600 | 1400 | Basal | 641 |
|  |  |  |  |  | 0.1R | 548 |
|  |  |  |  |  | 0.5R | 653 |
|  |  |  |  |  | 1R | 503 |
|  |  |  |  |  | 10R | 677 |
|  |  |  |  |  | 100R | 619 |
|  |  |  |  |  | **Total** | **3641** |
| data_with_p_values_MRT68921_conc | 3 | 1972 | 8700 | 6728 | 100M | 328 |
|  |  |  |  |  | 100R | 327 |
|  |  |  |  |  | 1M | 298 |
|  |  |  |  |  | Basal | 279 |
|  |  |  |  |  | 1MR | 277 |
|  |  |  |  |  | 100MR | 269 |
|  |  |  |  |  | **Total** | **1778** |

**Table S2**. Summary of experimental conditions used.

**Supplementary References**

[1] Beesabathuni NS, Park S, Shah PS. Quantitative and temporal measurement of dynamic autophagy rates. Autophagy [Internet]. 2022;00:1–20. Available from: https://doi.org/10.1080/15548627.2022.2117515.
